# Sulfated Fucogalactan From *Laminaria Japonica* Ameliorates β-Cell Failure by Attenuating Mitochondrial Dysfunction *via* SIRT1–PGC1-α Signaling Pathway Activation

**DOI:** 10.3389/fendo.2022.881256

**Published:** 2022-07-13

**Authors:** Nan Wu, Weihua Jin, Yuchen Zhao, Hong Wang, Sunyue He, Wenjing Zhang, Jiaqiang Zhou

**Affiliations:** ^1^ Department of Endocrinology and Metabolism, Sir Run Run Shaw Hospital, Zhejiang University School of Medicine, Hangzhou, China; ^2^ College of Biotechnology and Bioengineering, Zhejiang University of Technology, Hangzhou, China

**Keywords:** pancreatic β-cell failure, mitochondrial dysfunction, senescence, insulin exocytosis, SIRT1–PGC1-α

## Abstract

As mitochondrial metabolism is a major determinant of β-cell insulin secretion, mitochondrial dysfunction underlies β-cell failure and type 2 diabetes mellitus progression. An algal polysaccharide of *Laminaria japonica*, sulfated fucogalactan (SFG) displays various pharmacological effects in a variety of conditions, including metabolic disease. We investigated the protective effects of SFG against hydrogen peroxide (H_2_O_2_)-induced β-cell failure in MIN6 cells and islets. SFG significantly promoted the H_2_O_2_-inhibited proliferation in the cells and ameliorated their senescence, and potentiated β-cell function by regulating β-cell identity and the insulin exocytosis-related genes and proteins in H_2_O_2_-induced β-cells. SFG also attenuated mitochondrial dysfunction, including alterations in ATP content, mitochondrial respiratory chain genes and proteins expression, and reactive oxygen species and superoxide dismutase levels. Furthermore, SFG resulted in SIRT1–PGC1-α pathway activation and upregulated the downstream Nrf2 and Tfam. Taken together, the results show that SFG attenuates H_2_O_2_-induced β-cell failure by improving mitochondrial function *via* SIRT1–PGC1-α signaling pathway activation. Therefore, SFG is implicated as a potential agent for treating pancreatic β-cell failure.

## Introduction

Type 2 diabetes mellitus (T2DM) has become epidemic worldwide and is a chronic metabolic disease characterized by insulin resistance and β-cell failure. Nevertheless, insulin resistance does not cause T2DM unless there is concomitant β-cell failure (1). In the face of insulin resistance, euglycemia is maintained by augmenting existing β-cell capacity for the secretory response to glucose (2). In time, this compensatory phase eventually transitions to a decompensatory phase; β-cells lose the proliferative ability and senescent β-cells accumulate and undergo dedifferentiation, leading to the gradual deterioration of β-cell function (3, 4). Numerous studies have demonstrated that mitochondrial dysfunction is associated with β-cell failure, as the mitochondria play a crucial role in β-cells by controlling insulin secretion (5–7). Anello et al. demonstrated that compared with that from non-diabetic donors, pancreatic β-cells from T2DM patients exhibited markedly altered mitochondrial function and morphology (8). Silva et al. reported that β-cell-specific disruption of mitochondrial transcription factor A (Tfam), a protein essential for mitochondrial function, leads to suppressed glucose-stimulated insulin secretion (GSIS), reduced β-cell mass, and ultimately glucose intolerance in mice (9). Imeglimin is a new class of oral drugs developed for the treatment of T2DM. The underlying mechanism of Imeglimin may involve in attenuation of mitochondrial dysfunction. It contributes to better glycemic control in patients with T2DM when given in combination with metformin. This information led to the hypothesis that medicines or food products aimed at reversing mitochondrial dysfunction maybe a better potential strategy for treating β-cell failure.

Seaweed is commonly used as seasoning and foodstuff in many countries, particularly in Asia. The brown seaweed *Laminaria japonica* is the most important economic seaweed cultured in China and is consumed as a health food. *L. japonica* exhibits numerous biological activities, including anti-tumor, anti-microbial, antioxidative, antiviral, anti-aging, anti-fatigue, and anti-inflammatory effects (10–15). Recent studies have demonstrated that polysaccharides are the main bioactive components of *L. japonica*, and consist of alginate, laminarin, fucoidan, and different proportions of xylose, galactose, and glucuronic acid (16). Some bioactive polysaccharides exert protective effects against β-cell failure. For example, Li et al. found that ι-carrageenan tetrasaccharide (ιCT), a novel marine oligosaccharide prepared with the marine enzyme Cgi82A, inhibits islet β-cell apoptosis by upregulating GLP-1 (17). Zhang et al. reported that mulberry leaf polysaccharide significantly promoted β-cell regeneration and elevated insulin secretion in streptozotocin-induced diabetic rats (18). Yang et al. found that bee pollen polysaccharide from *Rosa rugosa* Thunb. (Rosaceae) regulates pancreatic β-cell proliferation and function (19). Therefore, based on the activity of other algae polysaccharides, we hypothesized that *L. japonica* polysaccharides might protect β-cell function.

In this study, we prepared sulfated fucogalactan (SFG) derived from *L. japonica* and evaluated its effects on β-cell failure using a hydrogen peroxide (H_2_O_2_)-induced MIN6 cell and islets model. We also investigated the underlying molecular mechanisms of SFG.

## Materials and Methods

### Reagents and Antibodies

H_2_O_2_ was purchased from Sigma-Aldrich (St. Louis, MO, USA). The insulin content assay kit was purchased from EZAssay (Shenzhen, China). The β-galactosidase (β-gal) activity kit was purchased from GenMed Scientific (Shanghai, China). The ATP assay kit was purchased from Beyotime (Shanghai, China). The reactive oxygen species (ROS) and superoxide dismutase (SOD) assay kits were purchased from Nanjing Jiancheng Bioengineering Institute (Nanjing, China).

The antibodies against Ki-67, γH2A.X, Kif5b, Snap25, SIRT1, PGC1-α, Tfam, and Nrf2 were purchased from Abcam (Cambridge, UK). Stx1, Aqp2, Atp5α, Uqcrc2, Mtco1, Sdhb and Ndufb8 were purchased from ABclonal (Wuhan, China). Kir6.2 was purchased from origene (Maryland, USA).

### Preparation of SFG

Fucoidan extracted from *L. japonica* was prepared according to a previous study (20). Then, 1 g fucoidan was degraded in 0.1 M HCl for 2 h at 80°C. The degradation solution was neutralized, concentrated, and precipitated by ethanol. Then, the precipitation was re-dissolved and degraded in 0.5 M HCl. The degradation solution was again neutralized, concentrated and precipitated by ethanol. The precipitation was re-dissolved, purified on a Bio Gel P10 column (2.6 × 100 cm) (Bio-Rad, CA, USA), and eluted with 0.2 M NH_4_HCO_3_ to obtain SFG. SFG powder was dissolved in phosphate-buffered saline (PBS) as stock solution with a concentration of 20 mg/ml. Polysaccharide composition and molecular weight (MW) were determined according to previous studies (21–23). In brief, 1-Phenyl-3-methyl-5-pyrazolone (PMP) is a common sugar derivatization agent used in high-performance liquid chromatography (HPLC). Polysaccharide composition was determined by PMP derivatization HPLC, including the total sugar content, fucose (Fuc) content, uronic acid (UA) content, sulfate content. The gel permeation chromatography–high-performance liquid chromatography (GPC-HPLC) analysis are used to estimate the MW.

### Cell Culture

The MIN6 cells were cultured in high-glucose Dulbecco’s modified Eagle’s medium containing 0.05% 2-mercaptoethanol, 15% embryonic stem-cell screened fetal bovine serum, 100 U/ml penicillin, and 100 μg/ml streptomycin at 37°C in a humidified atmosphere under 5% CO_2_. Cells were exposed to 125 μM H_2_O_2_ for 2 h. Then, the H_2_O_2_-containing medium was replaced with fresh medium containing 0, 25, 50, 100, or 200 μg/ml SFG for an additional 24 h before harvesting.

### Cell Viability Assay

The MIN6 cells were seeded in 96-well plates at a density of 1 × 10^4^ cells per well. Cells were treated with 125 μM H_2_O_2_ for 2 h, then replaced with fresh medium containing various concentrations of SFG and incubated for 24 h. After treatment, the culture supernatant was removed, and the cells were incubated in culture medium containing 10 μl Cell Counting Kit-8 (CCK-8, Yeasen Biotech, Shanghai, China) solution for 1.5 h at 37°C in the dark. The absorbance in each well was measured at 450 nm using a Multiskan GO microplate reader (Thermo Fisher Scientific, Waltham, MA, USA).

### GSIS Assay

The MIN6 cells were plated in 12-well plates at a density of 2 × 10^5^ cells per well. Cells were treated with 125 μM H_2_O_2_ for 2 h, then replaced with fresh medium containing various concentrations of SFG and incubated for 24 h. After treatment, the cells were incubated with glucose-free Krebs-Ringer bicarbonate buffer (KRBH, containing 0.1% BSA) for 30 min before 1 h incubation in KRBH buffer containing 2 mM or 25 mM glucose. The supernatant was collected, and the insulin content was measured by enzyme-linked immunosorbent assay. Results of insulin secretion were normalized by protein concentration and expressed as fold change related to control.

The method of isolating islets was referred to the previous study (O'Dowd et al., 2020). Islets were exposed to 125 μM H_2_O_2_ for 6 h and picked up to the fresh medium containing 50 and 100μg/ml SFG for an additional 24 h. Subsequently, islets were incubated with KRBH containing 0.1% BSA for 30 min and stimulated at 2.8 mM and 16.7 mM glucose KRBH buffer for 1 h separately. The supernatant was collected, for detecting insulin content of islets, the steps were the same as for the detection of MIN6 cells.

### Total Internal Reflection Fluorescence Microscopy

The MIN6 cells were plated in 35-mm MatTek imaging dishes(Cellvis, CA, USA) at a density of 4 × 10^5^ cells per well. After 18 h culture for adhesion, the cells were transiently transfected with VAMP2-pHluorin using Lipofectamine 3000 according to the manufacturer’s protocol. At 24 h after transfection, the cells were treated with 125 μM H_2_O_2_ for 2 h and the medium was replaced with fresh medium containing various concentrations of SFG for 24 h. The cells transfected with VAMP2-pHluorin were serum-starved in KRBH buffer for 1 h prior to microscopy. Throughout the imaging experiment, the cells were kept in an Air-Therm (WPI) temperature-regulated environmental chamber (Shanghai JingHong Laboratory Equipment Co., Shanghai, China) at 37°C. Basal insulin secretion state of cells were imaged for 2 min in KRBH containing 2 mM glucose. Then glucose stock (50 mM) was added to the edge of the MatTek dish on the microscope stage to reach a final concentration of 25mM glucose. The cells were imaged for 6 min in the glucose-stimulated state. TIRFM was performed using an Olympus objective-type IX-70 inverted microscope fitted with a 60×/1.45 NA TIRFM lens (Olympus, Center Valley, PA, USA) controlled by Andor iQ software (Andor Technologies, South Windsor, CT, USA) and detected with a back-illuminated Andor iXon 897 EMCCD (electron multiplying charge-coupled device) camera (512 × 512, 14 bit; Andor Technologies) (24).

### Senescence-Associated-β-Gal Assay

The MIN6 cells were plated in 12-well plates at a density of 2 × 10^5^ cells per well. Cells were treated with 125 μM H_2_O_2_ for 2 h, then replaced with fresh medium containing various concentrations of SFG and incubated for 24 h. Senescence was assessed by X-Gal staining for detecting β-gal activity with a commercially available kit after 24 h incubation in the fixative solution provided in the kit.

### Immunofluorescence Assay

The MIN6 cells were plated in 12-well plates at a density of 2 × 10^5^ cells per well. Cells were treated with 125 μM H_2_O_2_ for 2 h, then replaced with fresh medium containing various concentrations of SFG and incubated for 24 h. The MIN6 cells were fixed in 4% paraformaldehyde for 20 min, permeabilized with 0.2% Triton X-100/PBS for 15 min, and blocked with 5% BSA for 1 h. Cells on coverslips were incubated overnight at 4°C with the detection antibodies. After several washes in PBS, the fixed cells were incubated for 1 h with a goat anti-rabbit secondary antibody (Invitrogen, CA, USA; 1: 200) and counterstained for 5 min with DAPI. After mounting with fluorescence decay-resistant medium, the cells were observed and photographed under a confocal microscope (IX83-FV3000, Olympus, Tokyo, Japan).

### RNA Isolation and Quantitative Real-Time PCR

The MIN6 cells were plated in 6-well plates at a density of 4 × 10^5^ cells per well. Cells were treated with 125 μM H_2_O_2_ for 2 h, then replaced with fresh medium containing various concentrations of SFG and incubated for 24 h. RNA was isolated using AG RNAex Pro reagent and reversed-transcribed using an Evo M-MLV RT Premix kit (Accurate Biotechnology, Hunan, China) according to the manufacturers’ protocols. Real-time PCR was performed using the LightCycler 480 II system (Roche, Basel, Switzerland) and a SYBR Green Premix Pro Taq HS qPCR kit (Accurate Biotechnology, Hunan, China). After normalization to actin, the relative gene expression was determined using the comparative threshold cycle (2^−ΔΔCT^) method. The primer sequences are shown in the **Supplementary Data**.

### Western Blot

The MIN6 cells were plated in 6-well plates at a density of 4 × 10^5^ cells per well. Cells were treated with 125 μM H_2_O_2_ for 2 h, then replaced with fresh medium containing various concentrations of SFG and incubated for 24 h. The MIN6 cells were lysed with cell lysis buffer containing a protease inhibitor cocktail. Protein concentrations were determined by a bicinchoninic acid protein assay kit (FdBio Science, Hangzhou, China). Total protein extracts were denatured and separated by 10% or 12.5% sodium dodecyl sulfate–polyacrylamide gel electrophoresis. The membranes were incubated with primary antibodies for detection at 4°C overnight. Subsequently, the membranes were incubated with horseradish peroxidase-conjugated anti-rabbit or anti-mouse secondary antibody. The proteins were visualized using an enhanced chemiluminescence kit (FdBio Science, Hangzhou, China).

### ATP Assay

The MIN6 cells were plated in 6-well plates at a density of 4 × 10^5^ cells per well. Cells were treated with 125 μM H_2_O_2_ for 2 h, then replaced with fresh medium containing various concentrations of SFG and incubated for 24 h. The ATP levels were measured using an ATP assay kit. The method is based on a luciferase-luciferin reaction assay. The results were normalized by protein concentration.

### ROS Assay

The MIN6 cells were plated in 6-well plates at a density of 4 × 10^5^ cells per well. Cells were treated with 125 μM H_2_O_2_ for 2 h, then replaced with fresh medium containing various concentrations of SFG and incubated for 24 h. ROS production was identified using an ROS assay kit. The treated MIN6 cells were washed with PBS and incubated with DCFH-DA without light for 1 h at 37°C. The cells were washed in PBS three times and the fluorescence intensity was detected using a fluorescence microscope (Nikon Corporation, Tokyo, Japan).

### SOD Assay

The SOD activity in the cell medium was analyzed with SOD assay kit according to the manufacturer’s protocol. The absorbance at 450 nm was recorded using a microplate reader (Synergy H4, BioTek Instruments, Winooski, VT, USA). The results were normalized by protein concentration.

### Statistical Analysis

All experiments were repeated in triplicate. The results are expressed as the mean ± SEM. One-way analysis of variance (GraphPad Prism 7) was used for data comparisons within multiple groups. P < 0.05 was set as the threshold for statistical significance.

## Results

### Chemical Composition of SFG

**Figure 1:**
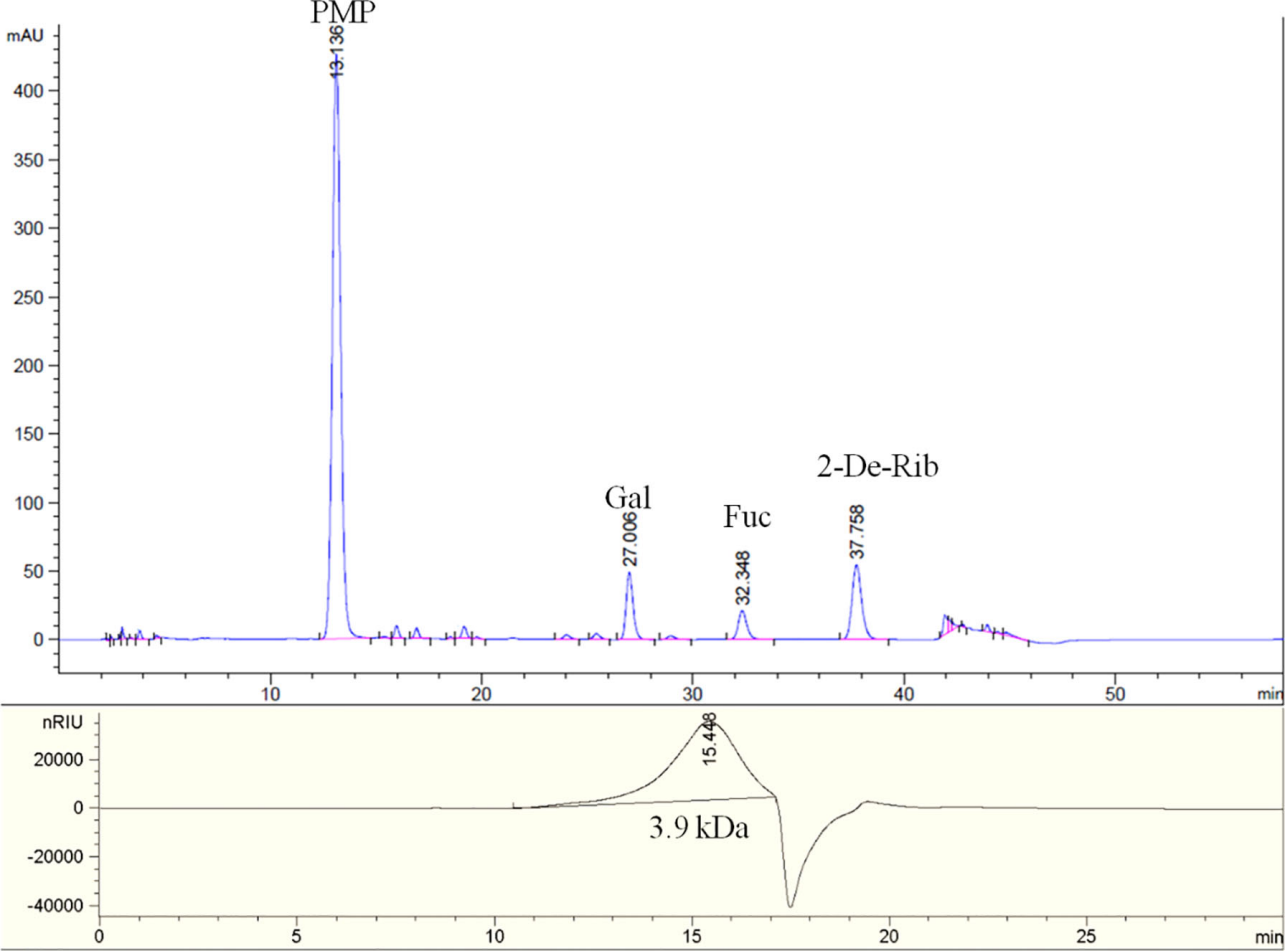
The PMP derivatization HPLC spectrum (top) and GPC-HPLC spectrum (bottom) of SFG.

The chemical composition analysis indicated that SFG contained 59.9% total sugar, 25.8% Fuc, 10.4% UA, and 13.1% sulfate. The molar ratio of monosaccharides of SFG was 1.25: 1 (galactose: Fuc), demonstrating that SFG it is mainly a sulfated fucogalactan (**Figure 1**). The GPC-HPLC analysis indicated that the average MW of SFG was approximately 3.9 kDa (**Figure 1**).

### SFG Increased β-Cell Viability and Improved Insulin Secretion Under H_2_O_2_ Exposure

**Figure 2:**
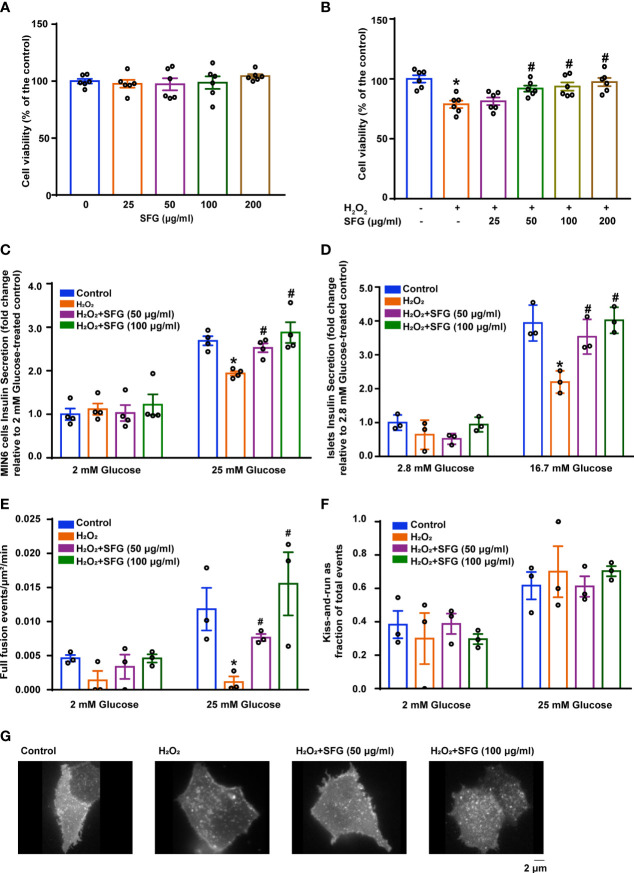
SFG increased H_2_O_2_-treated β-cell viability and improved insulin secretion. **(A)** Viability of MIN6 cells after SFG treatment in the absence of H_2_O_2_ exposure (n=6). **(B)** Viability of MIN6 cells exposed to H_2_O_2_ before SFG treatment (n=6). *P < 0.05 versus control; ^#^P < 0.05 versus H_2_O_2_-only group. **(C)** GSIS at 2 mM and 25 mM glucose in MIN6 cells (n=4). *P < 0.05 versus control; ^#^P < 0.05 versus H_2_O_2_-only group. **(D)** GSIS at 2.8 mM and 16.7 mM glucose in islets (n=3). *P < 0.05 versus control; ^#^P < 0.05 versus H_2_O_2_-only group. **(E)** SFG regulation of basal and glucose-stimulated insulin full fusion events. MIN6 cells transfected with VAMP2-pHluorin (n=3). *P < 0.05 versus control; ^#^P < 0.05 versus H_2_O_2_-only group. **(F)** The fraction of kiss-and-run events relative to total fusion events was quantified under basal and glucose-stimulated conditions. MIN6 cells transfected with VAMP2-pHluorin (n=3). **(G)** Representative MIN6 cells under glucose-stimulated state were observed under TIRFM.

The cytotoxicity of SFG was evaluated *in vitro*. After SFG (25–200 μg/ml) treatment, MIN6 cell viability was measured using the CCK-8 assay. There were no significant changes in cell viability after SFG treatment at any of the concentrations tested, showing that SFG had no obvious cytotoxic effects on the cells (**Figure 2A**). Compared with the untreated control cells, exposure to H_2_O_2_ inhibited MIN6 cell viability while treatment with 50–200 μg/ml SFG significantly alleviated this effect (P < 0.05) (**Figure 2B**). The effect of 50 and 100 μg/ml SFG was studied in the follow-up experiments.

The effect of SFG on MIN6 cell insulin secretion was investigated using the GSIS test. Cells that had been exposed to H_2_O_2_ had decreased insulin secretion in the presence of high glucose levels (25 mM) compared with that of the untreated control cells and SFG treatment enhanced insulin secretion (P < 0.05). Nevertheless, there was no difference under low-glucose stimulation (**Figure 2C**). Meanwhile, consisting with the results in MIN6 cells, GSIS test on islets isolated from C57BL/6 mice have also demonstrated that SFG treatment enhanced insulin secretion in the presence of high glucose levels (16.7 mM) (P < 0.05) (**Figure 2D**).The regulation of fusion pore dynamics is important for insulin secretion, as premature fusion pore closure is a likely mechanism by which insulin release is prevented. A recent study revealed two exocytosis modes: (1) full fusion, in which the exocytic vesicle and the plasma membrane fusion completely; and (2) kiss‐and‐run events, in which insulin granules release their contents through a transiently opened fusion pore (25). Here, H_2_O_2_-induced MIN6 cells had fewer glucose-stimulated full fusion events compared with the control cells, and SFG treatment significantly increased full fusion events (P < 0.05) (**Figure 2E**). There was no difference in the proportion of kiss-and-run fusion events between the treatment and control groups (**Figure 2F**). Representative MIN6 cells under glucose-stimulated state were observed under TIRFM (**Figure 2G**). Our findings demonstrate that H_2_O_2_ exposure results in significantly reduced insulin secretion in MIN6 cells, which is alleviated by SFG treatment.

### SFG Promoted the Inhibited Proliferation of H_2_O_2_-Induced MIN6 Cells and Ameliorated Their Senescence

**Figure 3:**
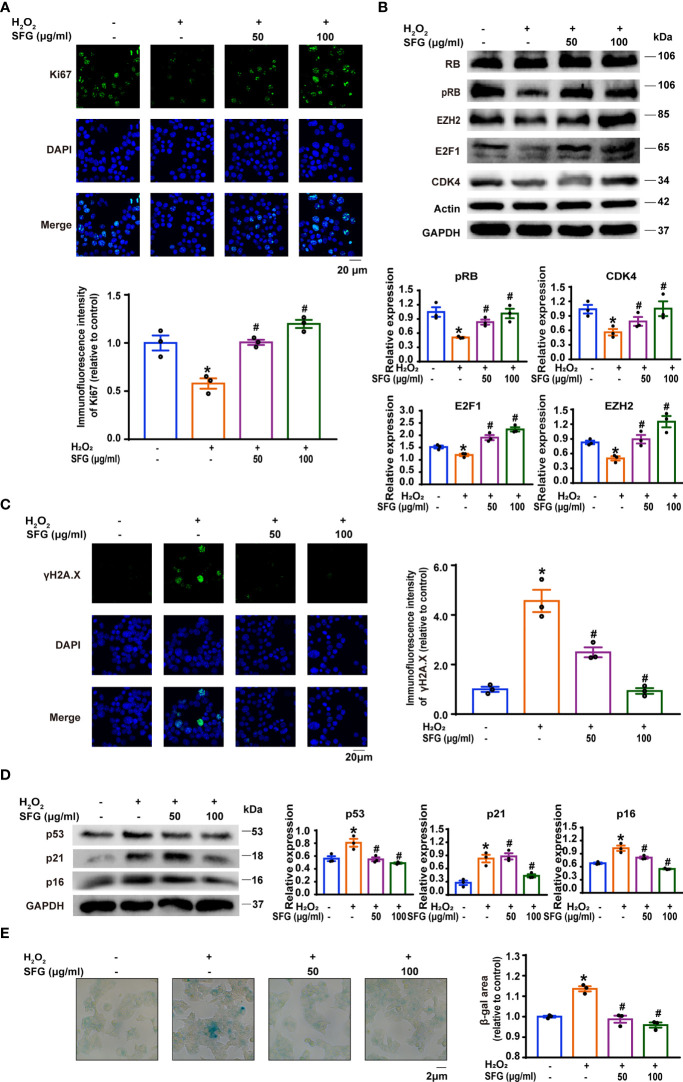
SFG promoted the inhibited proliferation of H_2_O_2_-treated MIN6 cells and ameliorated their senescence. **(A)** Immunofluorescence staining of Ki-67 expression in MIN6 cells (n=3). Quantitative analysis of Ki-67 expression was shown. *P < 0.05 versus control; ^#^P < 0.05 versus H_2_O_2_-only group. **(B)** Western blot analysis of the expression of the cell cycle regulator proteins RB, pRB, EZH2, E2F1, and CDK4 in MIN6 cells (n=3). GAPDH was used as a loading control for pRB, EZH2 and E2F1, actin was used as a loading control for CDK4. Densitometric analysis of the Western blot bands were shown. *P < 0.05 versus control; ^#^P < 0.05 versus H_2_O_2_-only group. **(C)** Immunofluorescence staining of γH2A.X in MIN6 cells (n=3). Quantitative analysis of γH2A.X expression was shown. *P < 0.05 versus control; #P < 0.05 versus H2O2-only group. **(D)** Western blot analysis of the expression of the senescence-associated proteins p16, p21, and p53 in MIN6 cells (n=3). GAPDH was used as a loading control. Densitometric analysis of the Western blot bands were shown. *P < 0.05 versus control; ^#^P < 0.05 versus H_2_O_2_-only group. **(E)** SA-β-gal staining in MIN6 cells (n=3). β-gal staining area analysis was shown. *P < 0.05 versus control; ^#^P < 0.05 versus H_2_O_2_-only group.

We studied the effect of SFG on MIN6 cell proliferation *via* immunofluorescence staining of Ki-67, a key cell proliferation marker (26). H_2_O_2_ exposure significantly inhibited Ki-67 expression in the cells and SFG eliminated this effect (P < 0.05) (**Figure 3A**). We examined the protein expression of the cell cycle regulators CDK4, RB, pRB, E2F1, and EZH2. Compared with the untreated control cells, H_2_O_2_-treated MIN6 cells had decreased CDK4, pRB, E2F1, and EZH2 protein expression, which was increased with the addition of SFG (P < 0.05) (**Figure 3B**). This indicates that SFG facilitated cell proliferation through cell cycle upregulation in H_2_O_2_-treated MIN6 cells.

We investigated whether SFG treatment would alleviate MIN6 cell senescence. The expression of senescence-related phenotypes was detected, including the senescence marker γH2A.X; the cellular senescence-associated proteins such as p16, p21, and p53; and SA-β-gal activity. Immunofluorescence staining showed that H_2_O_2_ increased γH2A.X accumulation in the cells as compared with the untreated control cells while SFG treatment rescued this effect (P < 0.05) (**Figure 3C**). Western blot confirmed that p16, p21, and p53 protein levels were greatly upregulated in the H_2_O_2_ group and that SFG treatment downregulated all three proteins (P < 0.05) (**Figure 3D**). Compared with the untreated control cells, H_2_O_2_-treated MIN6 cells had widespread and intense SA-β-gal-positive staining, while only sporadic staining was detected in the cells following SFG treatment (P < 0.05) (**Figure 3E**). Taken together, SFG promotes the inhibited proliferation in H_2_O_2_-treated MIN6 cells and ameliorates their senescence.

### SFG Potentiated β-Cell Function by Regulating β-Cell Identity and the Insulin Secretion-Related Genes and Proteins in H_2_O_2_-Treated MIN6 Cells

**Figure 4:**
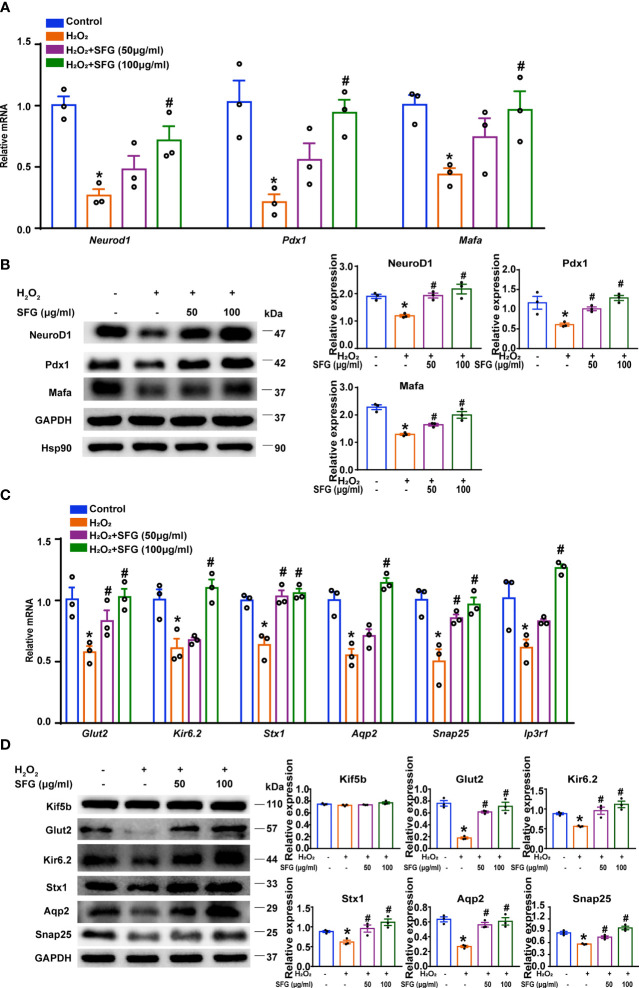
SFG potentiated β-cell function by regulating β-cell identity and the insulin secretion-related genes and proteins of H_2_O_2_-treated MIN6 cells. **(A)** *Neurod1*, *Mafa*, and *Pdx1* gene expression (n=3). *P < 0.05 versus control; ^#^P < 0.05 versus H_2_O_2_-only group. **(B)** Western blot of NeuroD1, Mafa, and Pdx1 (n=3). GAPDH was used as a loading control for NeuroD1 and Pdx1, Hsp90 was used as a loading control for Mafa. Densitometric analysis of the Western blot bands were shown. *P < 0.05 versus control; ^#^P < 0.05 versus H_2_O_2_-only group. **(C)** Gene expression of *Glut2*, *Snap25*, *Ip3r1*, *Aqp2*, *Stx1*, and *Kir6.2* (n=3). *P < 0.05 versus control; ^#^P < 0.05 versus H_2_O_2_-only group. **(D)** Western blot analysis of the expression of Glut2, Kir6.2, Stx1, Aqp2, and Snap25 in MIN6 cells (n=3). GAPDH was used as a loading control. Densitometric analysis of the Western blot bands were shown. *P < 0.05 versus control; ^#^P < 0.05 versus H_2_O_2_-only group.

We evaluated several genes and proteins that are essential in β-cells. NeuroD1, Mafa, and Pdx1 are critical for maintaining function in mature β-cells. The decreased expression of these key β-cell markers is associated with cellular dedifferentiation (27). In our study, *Neurod1*, *Mafa*, and *Pdx1* gene levels were dramatically downregulated in the H_2_O_2_ group while SFG treatment upregulated all three genes (P < 0.05) (**Figure 4A**). Western blot revealed a similar pattern for the *Neurod1*, *Mafa*, *Pdx1* mRNA levels (P < 0.05) (**Figure 4B**).

We then examined the genes and proteins crucial to insulin secretion. Glut2 controls glucose entry into cells and is key to maintaining glucose homeostasis (28). Kif5b is responsible for the transport of insulin-containing granules (29). *Glut2* mRNA levels was significantly reduced in H_2_O_2_-treated MIN6 cells and was upregulated by the addition of SFG (P < 0.05) (**Figure 4C**). Glut2 protein expression revealed a similar pattern for the *Glut2* mRNA levels (P < 0.05).While there was no difference in Kif5b protein expression (**Figure 4D**). We also evaluated some genes and proteins essential for vesicle fusion with the plasma membrane to release insulin. H_2_O_2_ resulted in significantly decreased *Snap25*, *Ip3r1*, *Aqp2*, *Stx1*, and *Kir6.2* mRNA levels, while SFG increased these mRNA expression (P < 0.05) (**Figure 4C**). Western blot revealed a similar pattern for the *Snap25, Aqp2, Stx1, and Kir6.2* mRNA levels (P < 0.05) (**Figure 4D**). These data indicate that SFG potentiates β-cell function in MIN6 cells by regulating β-cell identity and the insulin secretion-related genes and proteins.

### SFG Alleviated Mitochondrial Dysfunction and Decreased ROS Generation in H_2_O_2_-Treated MIN6 Cells

**Figure 5:**
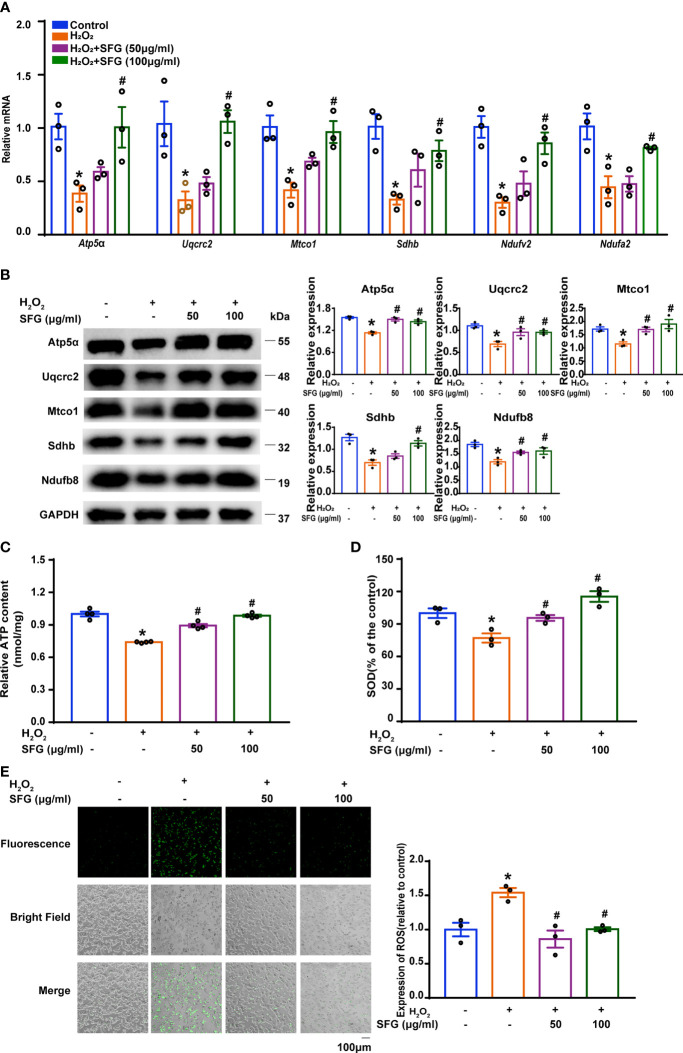
SFG alleviated mitochondrial dysfunction and decreased ROS generation in H_2_O_2_-treated MIN6 cells. **(A)** Gene expression of *Sdhb*, *Mtco1*, *Uqcrc2*, *Atp5a*, *Ndufv2*, and *Ndufa2* in MIN6 cells (n=3). *P < 0.05 versus control; ^#^P < 0.05 versus H_2_O_2_-only group. **(B)** Western blot analysis of the expression of Atp5α, Uqcrc2, Mtco1, SDHB, Ndufb8 in MIN6 cells (n=3). GAPDH was used as a loading control. Densitometric analysis of the Western blot bands were shown. *P < 0.05 versus control; ^#^P < 0.05 versus H_2_O_2_-only group. **(C)** ATP content in MIN6 cells (n=3). *P < 0.05 versus control; ^#^P < 0.05 versus H_2_O_2_-only group. **(D)** SOD levels in MIN6 cells (n=3). *P < 0.05 versus control; ^#^P < 0.05 versus H_2_O_2_-only group. **(E)** ROS levels in MIN6 cells (n=3). Quantitative analysis of ROS was shown. *P < 0.05 versus control; ^#^P < 0.05 versus H_2_O_2_-only group.

As the powerhouse of the cell, the mitochondria play a central role in maintaining β-cell health and function, which is involved in integrating and generating metabolic signals to control insulin secretion (5). Here, we explored the effect of SFG on mitochondrial function by detecting mitochondrial respiratory chain related proteins and genes and ATP content in MIN6 cells. H_2_O_2_-treated MIN6 cells had decreased levels of mitochondrial respiratory chain related proteins and mRNA, but SFG supplementation restored the decrease (P < 0.05) (**Figures 5A, B**). Meanwhile, the ATP content is decreased when exposed to H_2_O_2_, and increased when supplied with SFG (P < 0.05) (**Figure 5C**). The mitochondria are the primary site of ROS production and are susceptible to oxidative stress (30). Here, the H_2_O_2_-treated MIN6 cells had higher ROS levels and lower SOD levels than the untreated control cells. SFG markedly reduced ROS levels while significantly increasing SOD activity (P < 0.05) (**Figures 5D, E**). Our data indicate that H_2_O_2_ promotes mitochondrial dysfunction and ROS accumulation in MIN6 cells, leading to β-cell failure, and that SFG treatment alleviates these effects.

### SFG Counteracts the H_2_O_2_-Induced Negative Effect in MIN6 Cells *via* SIRT1–PGC1-*α* Signaling Pathway Activation

**Figure 6:**
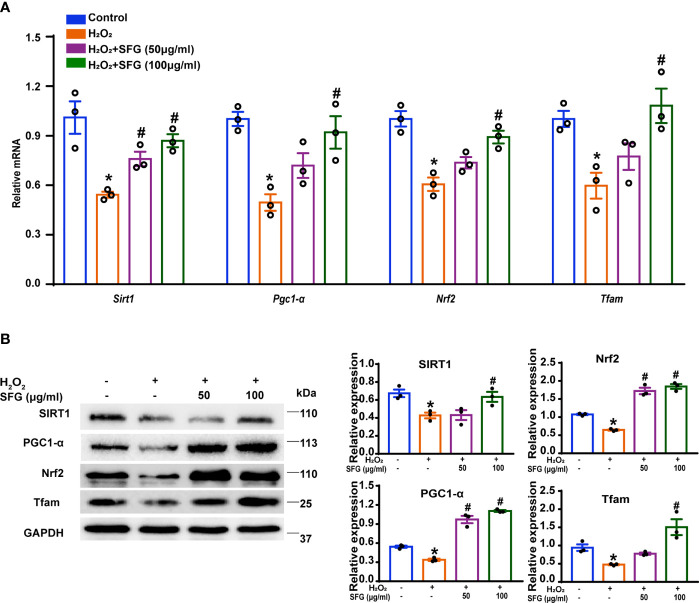
SFG counteracts the H_2_O_2_-induced negative effect on the PGC1-*α* signaling pathway in MIN6 cells. **(A)** *Sirt1*, *Pgc1a*, *Tfam*, and *Nrf2* gene expression (n=3). *P < 0.05 versus control; ^#^P < 0.05 versus H_2_O_2_-only group. **(B)** Western blot of SIRT1, PGC1-*α*, Tfam, and Nrf2 (n=3). GAPDH was used as a loading control. Densitometric analysis of the Western blot bands were shown. *P < 0.05 versus control; ^#^P < 0.05 versus H_2_O_2_-only group.

PGC1-*α* is a critical regulator of oxidative metabolism and is involved in maintaining mitochondrial biogenesis and function (31). SIRT1 acts as an upstream kinase and activates PGC1-*α* expression directly (32). In the present study, H_2_O_2_-treated MIN6 cells had markedly decreased *Sirt1* and *Pgc1-α* mRNA expression compared with the untreated control group, and SFG increased *Sirt1* and *Pgc1-*α mRNA expression (P < 0.05) (**Figure 6A**). PGC1-*α* exerts effects through the coactivation of many nuclear receptors and factors outside the nuclear receptor family. Nrf2 and Tfam are key targets of PGC1-α in mitochondrial biogenesis (32). *Nrf2* and *Tfam* mRNA expression was also downregulated in MIN6 cells following exposure to H_2_O_2_, and SFG supplementation upregulated their expression (P < 0.05) (**Figure 6A**). Western Blot revealed a similar pattern in the *Sirt1*, *Pgc1-a*, *Nrf2*, and *tfam* mRNA levels (P < 0.05) (**Figure 6B**).

## Discussion

The brown seaweed *L. japonica* is the most important economic seaweed and is consumed as a health food in China. It exhibits numerous biological activities, including that against metabolic disorders (33, 34). In the present study, we hypothesized that SFG protects MIN6 cells from H_2_O_2_-induced β-cell failure by attenuating mitochondrial dysfunction *via* SIRT1–PGC1-α signaling pathway activation.

One of the most common metabolic disorders worldwide, T2DM leads to damage to the heart, vasculature, eyes, kidneys, and nerves over time (35). β-Cell failure is believed to occur in T2DM development and progression, when the state of β-cell compensation for insulin resistance fails, resulting in the deterioration of β-cell function (2). Growing evidence demonstrates that β-cell senescence is associated with β-cell failure. β-Cells with senescence induced by aging and stress exhibit impaired insulin secretion response to glucose challenges (3, 36). Sone and Kagawa reported that β-cell senescence occurred in diet-induced T2DM mice and led to insufficient insulin release (36). Wang et al. demonstrated that Fischer rats, an established aging animal model, synthesized nearly half the amount of proinsulin upon high-glucose stimulation (37). Using oral ABT263 to remove senescent β-cells improved glucose metabolism and β-cell function in high-fat diet C57BL/6Crl mice (38). In our study, we verified that H_2_O_2_ induced GSIS impairment and insulin exocytosis dysfunction in β-cells, while SFG treatment restored the insulin secretion capacity of the cells. The exposure of MIN6 cells to H_2_O_2_ caused significantly decreased cell proliferation and induced senescence-like phenotypes, including alterations in DNA damage marker γH2A.X; the protein expression of related molecules, i.e., p16, p21, and p53; and SA-β-gal staining, while treatment with SFG could reverse these alterations. These data indicate that the addition of SFG restores insulin secretion capacity and ameliorates MIN6 cell senescence.

β-Cell dedifferentiation is recognized as a manifestation of impaired β-cell function (39). The dedifferentiated cells lose β-cell identity and exhibit defective insulin secretion, no longer controlling metabolic glucose homeostasis (40). The characteristics of β-cell dedifferentiation include reduced expression of key β-cell transcription factors such as NeuroD1, Mafa, and Pdx1 (41–43). Here, we verified that NeuroD1, Mafa, and Pdx1 genes and proteins expression in the MIN6 cells was downregulated following exposure to H_2_O_2_ and was upregulated with SFG treatment. Another indication of β-cell dysfunction is the altered dynamics of insulin release (39). Insulin release involves a sequence of events in β-cells: glucose enters the β-cells *via* Glut2, leading to Ca^2+^ influx. The rise in intracellular cytosolic Ca^2+^ is the primary mediator of insulin exocytosis, as it participates in integral events that include insulin secretory granule traffic to the plasma membrane, priming, docking, and exocytotic regulated release *via* SNARE complexes (28). In our study, H_2_O_2_ significantly decreased the expression of Glut2 and the insulin exocytosis-related genes and proteins while SFG treatment reversed the secretory dysfunction induced by H_2_O_2_. This indicates that SFG potentiates β-cell function by regulating β-cell identity and the insulin secretion-related genes and proteins of MIN6 cells against H_2_O_2_.

Mitochondrial dysfunction may be the underlying mechanism of β-cell failure. Ma et al. demonstrated that the overproduction of mitochondrial free radicals in β-cells was associated with decreased insulin secretion due to β-cell failure and diabetes (44). Song et al. reported that the inactivation of YY1, an important regulator of metabolic homeostasis, induced mitochondrial dysfunction and led to diabetes in mouse models (45). Therefore, an associative mechanism links mitochondrial dysfunction and T2DM development. In accordance with these findings, we show that SFG greatly alleviated abnormal mitochondrial functions, evidenced by the increased mitochondrial respiratory chain genes and proteins expression and ATP levels compared with the H_2_O_2_-treated MIN6 cells. In addition, the mitochondria are both the main sites of production and the main targets of ROS (30). As β-cells have unusually low antioxidant defense gene expression, they are particularly susceptible to cell damage induced by oxidative stress (30). By measuring ROS and antioxidant SOD levels, we show that SFG suppressed ROS production and increased SOD levels in H_2_O_2_-treated MIN6 cells, indicating that SFG reverses mitochondrial dysfunction by clearing intracellular ROS in H_2_O_2_-induced β-cell failure.

The best-studied Sirtuin protein family member, SIRT1 is a key molecule in bioenergy metabolism regulation. SIRT1 controls PGC1-α levels by deacetylating PGC1-α. PGC1-α is transcriptional co-activators that regulate activity of transcription factors including Nrf2 and Tfam and is known to play a crucial role in mitochondrial function (46). An increasing amount of evidence implicates PGC1-α as an important mediator of β-cell function. The expression of PGC1-α in β-cells is induced by extracellular signals including facilitators of GSIS, such as glucagon-like peptide-1(GLP-1) and cAMP, and stressors that impair β-cell function such as streptozotocin, glucocorticoids, obesity, cold exposure, and glucolipotoxicity (47–50). The pancreatic islets from T2DM patients have reduced *PGC1A* mRNA expression, which is associated with decreased insulin secretion (51). Moreover, silencing *PGC1A* leads to reduced insulin secretion in diabetic animals (51). Given the characterization of PGC1-α as critical regulators of mitochondrial function, we speculate that SFG may regulate the effects of SIRT1–PGC1-α signaling on β-cell function by alleviating mitochondrial dysfunction. In our study, H_2_O_2_ significantly decreased the gene and protein expression of SIRT1 and PGC1-α and the downstream of transcription factors such as Nrf2 and Tfam, while SFG treatment reversed this effect. These data show that the anti-mitochondrial dysfunction effect of SFG on MIN6 cells might be mediated through SIRT1–PGC1-α signaling pathway activation.

In conclusion, our results demonstrate that SFG protects β-cells against H_2_O_2_-induced cell failure possibly by attenuating mitochondrial dysfunction *via* SIRT1–PGC1-α signaling pathway activation. This finding could provide a mechanistic basis for SFG as a potential therapeutic strategy for protecting pancreatic β-cells.

## Data Availability Statement

The original contributions presented in the study are included in the article/**Supplementary Material**. Further inquiries can be directed to the corresponding authors.

## Ethics Statement

The animal study was reviewed and approved by Sir Run Shaw Hospital, Zhejiang University School of Medicine.

## Author Contributions

NW: Methodology, data acquisition, formal analysis, writing-original draft preparation, writing-reviewing and editing, project administration. WJ: Conceptualization, data acquisition, investigation, formal analysis, writing-original draft preparation. YZ: Data acquisition. HW: Data acquisition. SH: Data acquisition. WZ: Resources, data curation, formal analysis, writing-original draft preparation, supervision, funding acquisition, project administration. JZ: Data curation, writing-reviewing, editing, project administration, funding acquisition, and supervision. All authors contributed to the article and approved the submitted version.

## Funding

This work was supported by grants from the National Natural Science Foundation of China (No. 81870562 to JZ, No. 41906095 to WZ), the National Key Technology R&D Program of China (No. 2009BAI80B02 to JZ), the Zhejiang Provincial Natural Science Foundation of China (LZ22H070002 to JZ, No. LY22D060003 to WZ and LY19D060006 to WJ), and the Open Fund of Key Laboratory of Experimental Marine Biology, Chinese Academy of Sciences (No. KF2018NO2 to WJ).

## Conflict of Interest

The authors declare that the research was conducted in the absence of any commercial or financial relationships that could be construed as a potential conflict of interest.

## Publisher’s Note

All claims expressed in this article are solely those of the authors and do not necessarily represent those of their affiliated organizations, or those of the publisher, the editors and the reviewers. Any product that may be evaluated in this article, or claim that may be made by its manufacturer, is not guaranteed or endorsed by the publisher.
